# IL-6 and IL-10 Levels, Rather Than Viral Load and Neutralizing Antibody Titers, Determine the Fate of Patients With Severe Fever With Thrombocytopenia Syndrome Virus Infection in South Korea

**DOI:** 10.3389/fimmu.2021.711847

**Published:** 2021-08-17

**Authors:** Jeong Rae Yoo, Tae-Jin Kim, Sang Taek Heo, Kyung-Ah Hwang, Hyunjoo Oh, TaeHong Ha, Hye Kyung Ko, Seungjae Baek, Ju Eun Kim, Jun Hyeong Kim, Jiin Lee, Min Ji Kang, Mi Soo Yoo, Jung Mogg Kim, Kyung-Mi Lee, Keun Hwa Lee

**Affiliations:** ^1^Department of internal Medicine, Jeju National University College of Medicine, Jeju, South Korea; ^2^Department of Biochemistry, Korea University College of Medicine, Seoul, South Korea; ^3^Department of Research and Development, SML Genetree, Seoul, South Korea; ^4^Deparment of Microbiology, Hanyang University College of Medicine, Seoul, South Korea

**Keywords:** severe fever with thrombocytopenia syndrome, tick-borne viral diseases, IL-6, IL-10, South Korea

## Abstract

Severe fever with thrombocytopenia syndrome (SFTS) is a new tick-borne viral disease, and most SFTS virus (SFTSV) infections occur *via* bites from the tick *Haemaphysalis longicornis*; however, SFTSV transmission can also occur through close contact with an infected patient. SFTS is characterized by acute high fever, thrombocytopenia, leukopenia, elevated serum hepatic enzyme levels, gastrointestinal symptoms, and multiorgan failure and has a 16.2 to 30% mortality rate. In this study, we found that age, dyspnea rates, aspartate aminotransferase (AST), alanine aminotransferase (ALT), lactate dehydrogenase, multiorgan dysfunction score (MODS), viral load, IL-6 levels, and IL-10 levels were higher in patients with fatal disease than in patients with nonfatal disease during the initial clinical course of SFTS. In addition, we found that IL-6 and IL-10 levels, rather than viral load and neutralizing antibody titers, in patients with an SFTSV infection strongly correlated with outcomes (for severe disease with an ultimate outcome of recovery or death).

## Introduction

Severe fever with thrombocytopenia syndrome (SFTS), a new tick-borne viral disease with a high mortality rate, was first reported in China in 2009, South Korea in 2010, Japan in 2013, Vietnam in 2017, Myanmar in 2018, Taiwan in 2019, and Thailand and Pakistan in 2020 (1–8).

Most SFTSV infections occur *via* bites from the tick *Haemaphysalis longicornis*; however, SFTSV transmission can also occur through close contact with an infected patient (9).

SFTS is characterized by acute high fever, thrombocytopenia, leukopenia, elevated serum hepatic enzyme levels, gastrointestinal symptoms, and multiorgan failure and has a 16.2 to 30% mortality rate (1, 3, 6, 10).

In this study, we report the clinical and laboratory variables and clinical outcomes of confirmed SFTS patients with nonfatal and fatal disease from 2013 to 2019 in South Korea and show that age, dyspnea rates, aspartate aminotransferase (AST), alanine aminotransferase (ALT), lactate dehydrogenase, multiorgan dysfunction score (MODS), viral load, IL-6 levels, and IL-10 levels were higher in patients with fatal disease than in patients with nonfatal disease during the initial clinical course of SFTS.

In addition, we found that systemic IL-6 and IL-10 levels in patients with an SFTSV infection more strongly correlated with outcomes (for severe disease with an ultimate outcome of recovery or death) than did viral load and neutralizing antibodies.

## Materials And Methods

We confirmed 62 SFTS patients treated at a single tertiary hospital on Jeju Island from April 2013 to December 2019 (case fatality rate (CFR = 11.2%), and 54 SFTS patients were enrolled in the study (**Table 1**).

**Table 1:** Demographics and baseline characteristics of patients infected with SFTSV in Jeju, South Korea, from 2013 to 2019 (*n*=54).

Variables	All patients (*n* = 54)	Patients with nonfatal disease (*n* = 47)	Patients with fatal disease (*n* = 7)	*p*-value
Age ± SD (years)	62.8 ± 14.3	61.4 ± 14.4	72.0 ± 9.9	0.046
Sex, n (%)				
Men, n (%)	31 (58.5)	25 (54.3)	6 (85.7)	0.12
CCI ± SD	0.4 ± 0.7	0.3 ± 0.7	0.9 ± 0.9	0.04
Febrile sensation, n (%)	49 (92.5)	45 (97.8)	4 (57.1)	0.001
Myalgia, n (%)	21 (39.6)	25 (54.3)	6 (85.7)	0.02
Diarrhea, n (%)	22 (41.5)	19 (41.3)	3 (42.9)	0.03
Hemoptysis, n (%)	2 (3.8)	1 (2.2)	1 (14.3)	0.04
Dyspnea, n (%)	2 (3.8)	0 (0)	2 (28.6)	0.001
Mean time from symptom onset to diagnosis ± SD	5.6 ± 3.1	5.8 ± 3.2	5.4 ± 2.9	0.79
Mean body temperature ± SD (°C)	38.6 ± 0.8	38.6 ± 0.8	38.1 ± 0.9	0.04
ANC ± SD (cells/μL)	1,403.0 ± 1,307.1	1,373.2 ± 1,308.8	1,621.8 ± 1,395.5	0.67
Platelet count ± SD (/μL)	57,682.6 ± 28,924.2	5,963.0 ± 34,028.4	44,700.0 ±8,657.9	0.24
CRP ± SD (mg/dL)	0.9 ± 1.7	0.82 ± 1.5	1.8 ± 2.6	0.16
AST ± SD (IU/L)	182.6 ± 301.3	122.8 ± 140.7	575.6 ± 656.5	<0.001
ALT ± SD (IU/L)	77 ± 87.3	64.0 ± 55.2	162.3 ± 182.8	0.004
CK ± SD (IU/L)	1,069.6 ± 1,741.7	9,45.1 ± 1592.3	1,834.3 ± 2,496.0	0.21
LDH ± SD (IU/L)	1,083.6 ± 1,303.6	828.0 ± 631.7	3,042.7 ± 2,921.6	<0.001
MODS ± SD	4.43 ± 4.3	3.0 ± 1.8	13.7 ± 4.2	<0.001
Viral load ± SD (copies/mL)	98,022,220.9 ± 702,692,683.2	403,658.3 ± 795,822.0	725,570,123.6 ± 1,914,726,379.0	0.0019

Values are presented as the mean ± standard deviation. SFTSV, severe fever with thrombocytopenia syndrome virus; CCI, Charlson Comorbidity Index; ANC, absolute neutrophil count; CRP, c-reactive protein; AST, aspartate aminotransferase; ALT, alanine aminotransferase; CK, creatinine kinase; LDH, lactate dehydrogenase; MODS, multiorgan dysfunction score.

To investigate demographic, clinical, and laboratory variables, including SFTS viral loads (Ct value) and the levels of cytokines (obtained during the first visit to the hospital), we collected 155 serum samples from 54 patients (patients with nonfatal disease: *n* = 47, mean age: 61.4 ± 14.4; patients with fatal disease: *n* = 7, mean age: 72.0 ± 9.9, CFR = 12.96%). Laboratory variables were confirmed in the Laboratory Department of Jeju National University Hospital (**Table 1**). This study was approved by the Institutional Review Board (IRB) of Jeju National University Hospital (IRB file no. 2018-11-002).

For molecular diagnosis of SFTSV and measurements of viral load, RNA was extracted from stored patient serum (155 serum samples from 54 patients) using a QIAamp Viral RNA Mini kit (QIAGEN, Hilden, Germany). Real-time one-step RT-PCR was performed using an Ezplex^®^ SFTS virus Real-time PCR Kit (SMLGENETREE, South Korea) according to the manufacturer’s instructions. The patients were confirmed within one day in the hospital.

To characterize the effect of SFTSV infection on the production of serum cytokines in SFTS patients, interleukin-2 (IL-2), IL-4, IL-6, IL-10, IL-17A, tumor necrosis factor (TNF-α), and interferon-γ (IFN-γ) were measured using human Th1/Th2/Th17 CBA kits (BD Bioscience, San Diego, CA) according to the manufacturer’s instructions, with minor modifications. Sample acquisitions were performed with a FACS Canto II flow cytometer and analyzed by FCAP Array software version 3.0 (BD Bioscience, San Diego, CA).

All statistical analyses were performed using SPSS version 20.0 (IBM Corp., Armonk, NY, USA). *P* values < 0.05 indicated statistical significance. To compare the mean difference between patients with fatal and nonfatal disease, we usually used a two-samples t-test. When using this method, we checked some assumptions, such as normality, equal variance, and independence. In this case, the two groups had quite different sample sizes (*n *= 47 and *n *= 7), and the normality assumption for each group did not hold. We used a nonparametric two-sample t-test called the Wilcoxon-Mann-Whitney test (**Tables 1** and **2**).

**Table 2:** Comparison of cytokine concentrations between patients with nonfatal and fatal disease^a^.

Characteristic	Patients with nonfatal disease (*n *= 47)	Patients with fatal disease (*n* = 7)	*p*-value
IL-2	0.3 (0.0-2.3)	0.6 (0.0-2.1)	0.1352
IL-4	0.3 (0.0-2.7)	0.4 (0.0-1.0)	0.1765
IL-6	10.8 (0.0-34.8)	3,151.2 (17.2-15,103.8)	<0001
IL-10	8.8 (0.0-72.3)	42.9 (8.2-145.2)	0.0003
IL-17A	25.7 (0.0-120.2)	16.0 (1.5-43.3)	0.0896
IFN-γ	136.7 (0.0-679.6)	540.4 (0.0-3258.8)	0.3406
TNF	0.6 (0.0-7.6)	8.2 (0.0-51.3)	0.1521

Unit: pg/ml; ^a^Values are listed as the median and range unless otherwise noted.

## Results

We confirmed 62 SFTS patients treated at a single tertiary hospital on Jeju Island from April 2013 to December 2019 (case fatality rate (CFR) = 11.2%, and 54 SFTS patients (patients with nonfatal disease: *n* = 47, mean age: 61.4 ± 14.4; patients with fatal disease: *n* = 7, mean age: 72.0 ± 9.9, CFR = 12.96%) enrolled in the study (**Table 1**).

Among the 54 SFTS patients, age, dyspnea rates, body temperature, AST, ALT, LDH, MODS, viral load, IL-6 levels, and IL-10 levels were significantly associated with the outcomes of patients with SFTSV (**Tables 1**, **2**). Compared with patients with nonfatal disease, patients with fatal disease had higher age (*p*-value 0.046), dyspnea rates (0.001), AST (<0.001), ALT (0.004), LDH (<.0001), MODS (<.0001), viral load (0.0019), serum IL-6 levels (<.0001), and serum IL-10 levels (0.0003) and lower body temperature (0.04) during the initial clinical course of hospitalization (**Tables 1**, **2**). However, there were no statistically significant differences in plasma levels of IL-2, IL-4, IL-17A, TNF-α, and IFN-γ between patients with nonfatal and fatal disease (**Table 2**).

We also studied the kinetics of the viral load and cytokine levels and compared them with the titer of neutralizing antibodies, which was previously shown to differ between patients with fatal severe disease and patients with nonfatal severe disease (11).

In patients with nonfatal severe disease, the levels of IL-6 and IL-10 were lower, and the viral load was higher than those of patients with fatal severe disease and decreased over time. In our previous paper, we showed that the titer of neutralizing antibodies in patients with nonfatal severe disease increased over time, although one patient with nonfatal severe disease did not produce neutralizing antibodies, similar to patients with fatal severe disease (**Tables 3-1**, **3-2**).

**Table 3-1:** Comparison of IL-6 and IL-10 concentrations, viral load, and neutralizing antibody titers between patients with fatal severe disease and patients with nonfatal severe disease.

Patients	Age (years) /Sex	*Date	Severity	Outcome	IL-6	IL-10	Viral load (copies/mL)	FRNT50 titer (11)
P01-13	73/M	May-16-13	Severe	Death	2622.3	10.9	897.242.38	6.569
P04-13	63/M	Jun-13-13	Severe	Death	117.2	8.2	< 100	6.222
P16-15	74/M	Jun-10-15	Severe	Death	61.4	45.6	15,527.11	0
P49-18	81/F	Jun-12-18	Severe	Recovered	22.2	40.8	1,222,514.26	3.401
P64-19	64/M	Aug-12-19	Severe	Recovered	5.6	5.6	48,766.65	0
P66-19	60/F	Aug-28-19	Severe	Recovered	3.9	3.8	798.839.84	10.25
P70-19	70/F	Oct-11-19	Severe	Recovered	7.1	7.1	101.288.97	13.53

Unit: pg/ml, *Date: The hospitalization and sampling date; P, positive; N, negative; FRNT50, 50% focus reduction neutralization test.

**Table 3-2:** Kinetics of IL-6 and IL-10 concentrations, viral load, and neutralizing antibody titers in patients with nonfatal severe disease.

Patients	Age (years) /Sex	Date	Severity	Outcome	IL-6	IL-10	Viral load (copies/mL)	FRNT50 titer (11)
P49-18	81/F	*Jun-12-18	Severe	Recovered	22.2	40.8	1,222,514.26	3.401
		Jun-14-18			23.5	28.7	18,307.96	2.171
		Jun-16-18			18.8	6.7	4,798.72	0.6024
		Jun-18-18			13	2.2	2,785.74	9.89
P64-19	64/M	*Aug-12-19	Severe	Recovered	5.6	5.6	48,766.65	0
		Aug-14-19			2.5	1.5	3,266.81	0
		Aug-16-19			2.4	1	1,217.66	0
P66-19	60/F	*Aug-28-19	Severe	Recovered	3.9	3.8	798,839.84	10.25
		Aug-30-19			3.2	2.6	34,353.24	5.857
P70-19	70/F	*Oct-11-19	Severe	Recovered	7.1	7.1	101,288.97	13.53
		Oct-22-19			23.6	2.9	Undetermined	34.82

Unit: pg/ml, *The hospitalization and sampling date; P, positive; N, negative; FRNT50, 50% focus reduction neutralization test.

## Discussion

IL-6, a proinflammatory cytokine, is essential for escalating the cell response to control persistent viral infection, and expression of IL-10, an important anti-inflammatory cytokine, is significantly elevated in SFTS patients, especially in patients with fatal disease.

The overproduction of IL-6 and IL-10 can create a cytokine storm, which is considered to contribute to the pathology of SFTS (12, 13).

In this study, high levels of IL-6 and IL-10 and high viral loads were found to coexist in patients with fatal and nonfatal disease.

In addition, IL-6 and IL-10 levels were higher in patients with fatal severe disease than in patients with nonfatal severe disease, and the levels of these cytokines were both decreased in patients with nonfatal severe disease.

The viral load was higher in patients with nonfatal severe disease than in patients with fatal severe disease at the first visit to the hospital and decreased over time. The titers of neutralizing antibodies for some patients with nonfatal severe disease was lower than that of patients with fatal severe disease at the first visit to the hospital but increased over time. However, one patient did not produce neutralizing antibodies such as a patient with fatal severe disease.

Therefore, we suggest that IL-6 and IL-10 determine the fate of patients (for severe disease with an ultimate outcome of recovery or death) more than viral load and the titer of neutralizing antibodies.

The limitations of our study include the relatively small number of patients studied (*n *= 54). However, this is a rigorous prospective study that took 7 years (from 2013 to 2019) in a representative hospital for the treatment of SFTS on Jeju Island, South Korea.

In summary, we reported that the levels of serum IL-6 and IL-10 were elevated in patients with fatal severe disease, while the levels these cytokines decreased in patients with nonfatal severe disease. This suggests that IL-6 and IL-10, rather than viral load and the titer of neutralizing antibodies, play an important role in determining the fate of patients.

## Data Availability Statement

The original contributions presented in the study are included in the article/supplementary material. Further inquiries can be directed to the corresponding authors.

## Ethics Statement

This study was approved by the Institutional Review Board (IRB) of Jeju National University Hospital (IRB file no. 2018-11-002). The patients/participants provided their written informed consent to participate in this study.

## Author Contributions 

Conceptualization: KL. Methodology: KL, K-ML, JY, SH, TK, K-AH, HO, HK, SB, JEK, JHK, JL, MK, and TH. Supervision and validation: KL and K-ML. Formal analysis: KL, K-ML, JY, SH, TK, and K-AH. Funding acquisition: KL and K-ML. Data curation: KL and K-ML. Writing-original draft: KL, K-ML, JY, and TK. Writing-review and editing: KL and K-ML. All authors contributed to the article and approved the submitted version.

## Funding

This work was supported by the National Research Foundation of Korea (NRF), the Ministry of Science, ICT, and Future Planning of South Korea (grant number: NRF-2020R1A2C2103061), and research funding from Hanyang University (HY-2020), and we thank S.Y. Bae for reviewing this paper.

## Conflict of Interest

K-AH was employed by SML Genetree.

The remaining authors declare that the research was conducted in the absence of any commercial or financial relationships that could be construed as a potential conflict of interest.

## Publisher’s Note

All claims expressed in this article are solely those of the authors and do not necessarily represent those of their affiliated organizations, or those of the publisher, the editors and the reviewers. Any product that may be evaluated in this article, or claim that may be made by its manufacturer, is not guaranteed or endorsed by the publisher.
